# A Dictionary of Practical Medicine, &c.

**Published:** 1845-04

**Authors:** 


*A Dictionary of Practical Medicine, &c.* By JAMES COPLAND, M. D. F. R. S. *Edited by* CHARLES A. LEE, M. D.

We have received from the publishers, Harper & Brother, part IV. of this valuable book.

